# The Effects of Renal Nerve Denervation on Blood Pressure and Target Organs in Different Hypertensive Rat Models

**DOI:** 10.1155/2021/8615253

**Published:** 2021-04-04

**Authors:** Demin Liu, Jing Wang, Haijuan Hu, Guoqiang Gu, Rui Ding, Ruiqin Xie, Wei Cui

**Affiliations:** Department of Cardiology, The Second Hospital of Hebei Medical University, Shijiazhuang, Hebei, China

## Abstract

**Background:**

Hypertension contributes to the progression of cardiac remodeling and renal damage. In turn, renal sympathetic hyperactivation showed elevated sympathetic nervous system activity and led to blood pressure increase in certain patients. The purpose of this study was to observe the effect of renal nerve denervation on blood pressure and target organ changes in two hypertensive rat models.

**Methods:**

Hypertensive rats were randomly divided into a renal denervation (RDN) group and sham operation group. Wistar–Kyoto (WKY) rats of the same age were set as the baseline control group. In the secondary hypertension model, SD rats were randomly divided into five groups. Blood pressure and bodyweight were measured every week until they were euthanized.

**Results:**

The two rat models underwent RDN at key timepoints. At these timepoints, the hearts and kidneys were collected for norepinephrine and angiotensin II measurements and histological analysis.

**Conclusion:**

RDN performed before development of hypertension showed a significant antihypertensive effect on the secondary hypertension model.

## 1. Background

Hypertension is associated with increased sympathetic activation, possibly contributing to the progression of cardiac remodeling and renal damage [1]. Although medical therapy could reduce blood pressure and the incidence of cardiovascular events in hypertensive patients, some patients still cannot benefit from medical therapy [2]. Renal sympathetic denervation (RDN) decreases sympathetic renal efferent and afferent nerve activities. RDN as an interventional approach to target elevated sympathetic nervous system activity has received considerable interest, as it has led to blood pressure (BP) reduction in certain patients with uncontrolled hypertension [3, 4]. There is mixed clinical evidence supporting RDN as an effective antihypertensive treatment. Several randomized controlled trials [5–7] support the safety and efficacy of the procedure, but some smaller studies, as well as the randomized, sham-controlled SYMPLICITY HTN-3 trial, failed to suggest the superiority of RDN when compared with medical treatment alone [8]. The underlying pathophysiologic concept, though, is strongly supported by various experimental models of hypertension [9–11]. Surgical RDN in obese, spontaneously hypertensive rats prevented the progressive increase in BP and progression of renal injury and cardiac remodeling [12]. According to different pathogenesis of hypertension, there are two main types of hypertension—primary hypertension and secondary hypertension. A suitable, hypertensive animal model with long-term follow-up would greatly help to further assess BP effects and other surrogate markers of efficacy in detail, such as histological RDN and renal norepinephrine content [13]. Therefore, the purpose of this study is to observe the effect of RDN on the blood pressure and target organ changes in the two different hypertensive rat models.

## 2. Methods

### 2.1. Animals

Male Sprague–Dawley (SD) rats (weight: 200–220 g) were provided by Hebei Medical University Laboratory Animal Center. The rats were randomly divided into five groups (all of equal sample size, *n* = 20): control group, sham group, two-kidney one-clip (2K1C), two-kidney one-clip plus renal denervation (2K1C plus RDN), and two-kidney one-clip for 6 weeks plus renal denervation (2K1C plus 6RDN). SD rats were randomly sacrificed at the first, seventh, fourteenth, and eighteenth weeks after the 2K1C surgery in the five different groups. Twelve-week-old spontaneously hypertensive rats (SHR, *n* = 24) were randomly divided into a renal denervation group (RDN group) and sham operation group (sham group). Wistar–Kyoto (WKY) rats of the same age (*n* = 12) were set as the baseline control group. SHR rats, SD rats, and WKY rats were hosted in clean level environment, with light/dark cycle 12/12 h, a relative humidity of 50%–60%, the ambient temperature 22°C–25°C, 4-5 rats per cage, and free access to food and water. Four SHR rats were randomly sacrificed at one week, eight weeks, and twelve weeks after surgery in three different groups. BP and bodyweight (g) were measured before surgery and every week after surgery till the day before euthanasia in the two rat models. The two rat models underwent RDN at the key timepoints with the application of surgical resection and the chemical ablation method (using a solution of 10% phenol in ethanol). All experimental procedures were approved by the Ethics Committee of the Second Hospital of Hebei Medical University. The use and care of the animals complied with institutional guidelines.

#### 2.1.1. Systolic Blood Pressure (SBP) Measurements

In all rats, systolic blood pressure (SBP) was measured noninvasively using the tail-cuff method (LE 5001; PanLab, Barcelona, Spain), as described previously [14]. The SBP values were the average of three blood pressure measurements that were within 10 mmHg of each other. SBP was monitored throughout treatment, with the last measurement obtained 6 h before rats were killed.

#### 2.1.2. Renal Nerve Denervation

Both kidneys were approached through medial laparotomy and retroperitoneal incisions under general anesthesia with 1.5%–2.5% isoflurane. Both kidneys were surgically denervated by cutting all visible nerves around the renal hilus and by stripping approximately 2–4 mm of the adventitia from the renal artery. The area was then moistened with a phenol/ethanol solution for 20 minutes. A sham surgical procedure, with kidney exposure and no intervention, was performed in all other animals (SD-sham, SHR-sham, and WKY control rats) [15].

#### 2.1.3. 2K1C Renovascular Hypertension

The rats were placed in the left lateral position under general anesthesia with 1.5%–2.5% isoflurane. To minimize the invasive approach to the kidney, an incision was made in the dorsal midline. The traction of the erector spina muscles exposed the kidney, thereby allowing identification of the renal arteries and veins. A silver clip with an internal diameter of 0.25 mm was placed around the left renal artery, resulting in partial occlusion of the renal perfusion. After placing the clip, the flank incision was sutured. Immediately after the surgery, the animals received an appropriate dose of antibiotic (160,000 units of penicillin per rat, intramuscular). In the 2K1C plus RDN group, after the above operations, we then carried out the renal nerve denervation operation. In the sham group, the kidney was approached using the same mechanism as in the 2K1C group. The kidney was exposed, but the silver clip was not placed.

#### 2.1.4. Cardiac LVMI Measurements

The rats were euthanized, and their hearts were collected. The left ventricle (LV) was separated and weighed. The ratio of LV weight to bodyweight was calculated as the left ventricular mass index (LVMI).

#### 2.1.5. Enzyme-Linked Immunosorbent Assay

Venous blood samples were collected at the key timepoints (SHR: weeks 1, 8, and 12; SD: weeks 1, 7, 14, and 18). In SHR rats, the concentrations of NE and Ang II were measured at 1 week, 8 weeks, and 12 weeks. In SD rats, the concentrations of NE and Ang II were measured at 1 week, 7 weeks, 14 weeks, and 18 weeks postrenal nerve denervation, respectively. Plasma was obtained by centrifuging whole blood at 4°C (at 3000 rpm for 10 minutes); this plasma sample was stored at −80°C until ELISA testing was performed. Norepinephrine (NE) and angiotensin II (Ang II) levels at week ten were tested using ELISA kits. All procedures were in accordance with the instruction books (Cloud-Clone Corp CEA907Ge and CEA005Ra).

#### 2.1.6. Histology

The heart and kidney samples were harvested at the key timepoints. The left atrium (LA) tissue was separated from the heart and was fixed with 4% paraformaldehyde and then embedded in paraffin. The ventricle was cut transversely into two portions. One of the ventricle specimens was embedded in paraffin, and the comprising apex was stored at −80°C. Sections cut from the heart were stained with hematoxylin and eosin (H&E) reagent. For bilateral kidneys, the procedures were the same. Sections cut from paraffin blocks were stained with H&E. Five sections per sample were randomly selected, and five microscopic fields (×400) per section were selected for analysis. The collagen volume fraction (CVF, expressed as the percentage of the fibrosis area) was used to evaluate the extents of fibrosis by the ImagePro Plus 6.0 software, as described previously.

#### 2.1.7. Western Blotting

Membranous protein extraction from LV and kidney tissue was performed using the Mem-PER Membrane Protein Extraction Reagent Kit (Pierce Chemical, Rockford, IL, USA) according to the manufacturer's instructions. Brief, LV and kidney tissue were suspended in 1 mL ice-cold RIPA lysis buffer (50 mmol/L Tris-HCl, pH 7.4, 150 mmol/L NaCl, 0.25% sodium deoxycholate, 0.1% Nonidet P-40, and 0.1% sodium dodecyl sulphate (SDS), 1 mmol/L EDTA, 1 mmol/L Na_3_VO_4_, 1 mmol/L dithiothreitol, and 1 mmol/L phenylmethylsulphonyl fluoride) and homogenized for 1 min in a PRO tissue homogenizer (PRO Scientific, Shelton, CT, USA) at the highest setting. After centrifugation at 8000 g for 10 min, the protein concentration was determined using a modified Lowry protein assay. Equal membranous or total protein samples were electrophoresed on 12% SDS-polyacrylamide gels and then transferred onto polyvinylidene difluoride membranes. Membranes were incubated with appropriate dilutions of anticollagen I (1 : 1000, Abcam, USA), anticollagen III (1 : 1000, Abcam, USA), antimyosin heavy chain (1 : 2000, Abcam, USA), and anti-*β*-actin (1 : 1000, Santa Cruz, USA) antibodies overnight at 4°C. Samples were incubated in the presence of an alkaline phosphate secondary antibody for 1 h at room temperature in TTBS (500 mmol/L NaCl, 0.05% Tween 20, 50 mmol/L Tris-HCl, and pH 7.5) and processed for chemiluminescent detection according to the manufacturer's instructions (Santa Cruz Biotechnology). All bands were evaluated by densitometry with Quantity One V4.6.2 software (BioRad, Hercules, CA, USA). Bands of interest were normalized against *β*-actin, and data are presented as relative density ratios.

#### 2.1.8. Statistics

Continuous data are presented as mean ± standard error. For two group comparisons, data were analyzed with *t*-tests. For multiple comparisons, data were analyzed by a one-way analysis of variance (ANOVA) test followed by a least significant difference test. Repeated measures at multiple timepoints were analyzed by repeated measures ANOVA tests. SPSS 13.0 software was used to perform statistical analyses. A *p* value of *p* < 0.05 was considered to be statistically significant.

## 3. Results

### 3.1. Effect of RDN on Bodyweight

#### 3.1.1. Effect of RDN on Bodyweight in SHRs

**Figure 1:**
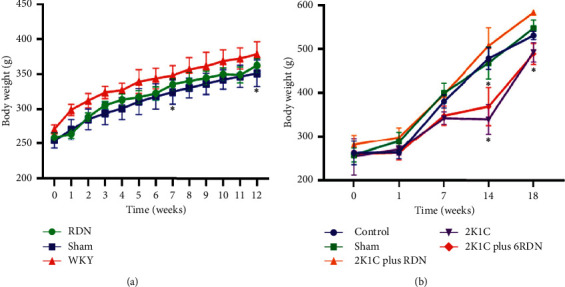
The comparison of bodyweight in SHR and SD rats. (a) The comparison of bodyweight in SHR (*n* = 24). ^*∗*^*p* < 0.05 vs. the sham group. (b) The comparison of bodyweight in SD rats (*n* = 100). ^*∗*^*p* < 0.05 vs. the sham group.

In the SHR rats, there was no significant difference in bodyweight between the RDN group and sham group at the corresponding point of time. However, the bodyweights of these two groups were significantly lower than those of the WKY group (*p* < 0.05) (Figure 1(a)).

#### 3.1.2. Effect of RDN on Bodyweight in SDs

Among all study groups in the SD rats, there were no differences in baseline bodyweight and weights resulting in one week following the 2K1C surgery (*p* > 0.05). At the seventh, fourteenth, and eighteenth weeks, the increase of bodyweight in the sham group and 2K1C plus RDN group exhibited no significant differences. However, the bodyweights of the 2K1C rats were significantly lower than those of the sham group; similarly, significant differences in bodyweight were seen between the 2K1C plus 6RDN group and sham group (*p* < 0.05). No significant differences in bodyweight were seen between the 2K1C group and 2K1C plus 6RDN group (*p* > 0.05), as shown in Figure 1(b).

### 3.2. Systolic Blood Pressure (SBP) and Neurohumoral Activation in SHRs and SDs

#### 3.2.1. SBP and Neurohumoral Activation in SHRs

**Figure 2:**
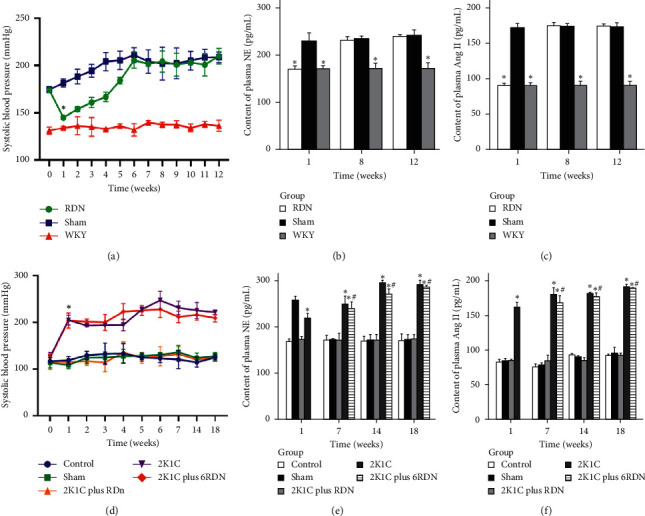
The comparison of systolic blood pressure and neurohumoral activation in SHR and SD rats. ^*∗*^*p* < 0.05 versus the control group, sham group, and 2K1C plus RDN group, ^#^*p* > 0.05 versus the 2K1C group.

There were no statistical differences in the SBP between the RDN and sham groups before surgery. However, the SBP in the RDN and sham groups was significantly higher than that in the WKY group (*p* < 0.05). The SBP of the RDN group was significantly lower than that of the sham group from week one through five following surgery (*p* < 0.05). There were no statistical differences in SBP between the RDN and sham groups after the sixth week (*p* > 0.05). The SBP of the sham group remained at a high level, and there was no significant change after the third week following surgery (*p* > 0.05). The SBP of the RDN and sham groups was higher than that of the WKY group at the preset time (*p* < 0.05) (Figure 2(a)). NE and Ang II plasma concentrations (Figures 2(b) and 22(c)) increased in SHR rats. The RDN procedure significantly reduced the concentrations of NE and Ang II in the plasma during the first week. However, the decline disappeared at the 8th week during which no difference was observed between the sham and RDN groups (*p* > 0.05).

#### 3.2.2. Blood Pressure (SBP) and Neurohumoral Activation in SDs

There were no differences in the baseline blood pressure among all study groups (*p* > 0.05). At the time of the experiment, the SBP in the sham group and 2K1C plus RDN group had no significant differences (*p* > 0.05). The SBP in the 2K1C group was significantly higher than that of the sham group (*p* < 0.05). At the seventh, fourteenth, and eighteenth weeks, no significant differences between the 2K1C and 2K1C plus 6RDN groups were observed (*p* > 0.05). Despite the SBP of the 2K1C plus 6RDN group being lower than that of the 2K1C group at the seventh, fourteenth, and eighteenth weeks (231.22 ± 14.23 mmHg vs. 211.56 ± 12.25 mmHg, 211.56 ± 12.25 mmHg vs. 216.11 ± 11.48 mmHg, and 221.22 ± 5.04 mmHg vs. 212.33 ± 6.17 mmHg, respectively), there was no significant difference between them (Figure 2(d)).

At the seventh week, the NE and Ang II levels of the 2K1C group had increased compared to the sham group (*p* > 0.05). Compared with the 2K1C group, RDN could have significantly decreased the concentrations of the NE and Ang II if the rats underwent the RDN and 2K1C procedures simultaneously. Compared with the 2K1C group, the concentrations of NE and Ang II in the 2K1C plus 6RDN group decreased; however, no significant differences were observed between the two groups (*p* > 0.05). At the eighteenth week, the levels of NE and Ang II in each group were consistent with the levels observed at the fourteenth week (Figures 2(e) and 2(f)).

### 3.3. Effect of RDN on Cardiac Remodeling

#### 3.3.1. LVMI Measurement

**Figure 3:**
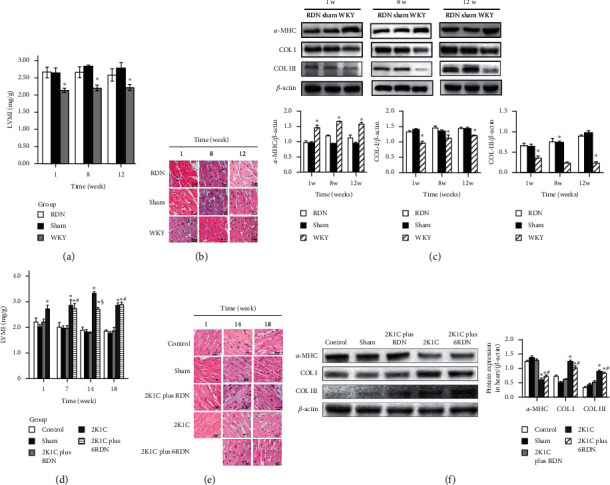
(a) Left ventricular mass index measurement in SHRs. ^*∗*^*p* < 0.05 versus the sham group (*n* = 12) at the same time. (b) Cardiomyocytes stained with H&E in SHRs. (c). The expression and quantification of *α*-MHC, Col I, and Col III protein in left ventricular of SHRs. ^*∗*^*p* < 0.05 versus the sham group (*n* = 5) at the same time. (d) Left ventricular mass index measurement in SDs. ^*∗*^*p* < 0.05 versus the control group (*n* = 5), sham group (*n* = 5), and 2K1C plus RDN group (*n* = 5); ^§^*p* < 0.05 versus the 2K1C group (*n* = 5); ^#^*p* > 0.05 versus the 2K1C group. (d) Cardiomyocytes stained with H&E in SDs. (e) Cardiomyocytes stained with H&E in SDs. (f) The protein expression and quantification of *α*-MHC, Col I, and Col III protein in left ventricular of SDs. ^*∗*^*p* < 0.05 versus the control group, sham group, and 2K1C plus RDN group; ^#^*p* > 0.05 versus the 2K1C group.

*(1) LVMI measurement in SHRs*. There were no statistical differences in LVMI measurements between the RDN group and sham group at the first, eighth, and twelfth weeks after surgery (*p* > 0.05). However, the LVMI was significantly lower in the WKY than in RDN and sham groups (*p* < 0.05) (Figure 3(a)).

*(2) LVMI measurement in SDs*. LVMI measurements in the 2K1C group significantly increased at the fourteenth and eighteenth weeks. Compared with the 2K1C group, the RDN group exhibited decreased LVMI measurements when the rats underwent the RDN and 2K1C procedures simultaneously (*p* < 0.05). However, compared with the 2K1C group, RDN did not play a role in the LVMI measurements in the 2K1C plus 6RDN group (*p* > 0.05). Similar results were observed at the eighteenth week in each group (Figure 3(d)).

*(3) Myocardial cell histology morphology and protein expression*. In both rat models, different degrees of damage of the myocardial cells were present, compared to the control group, which did not exhibit these traits. Some degrees of damage present included myocardial hypertrophy, disordered, myocardial interstitial edema, and partial myocardial cell lysis (with clear surrounding structures). Furthermore, RDN did not significantly attenuate cardiac hypertrophy and fibrosis formation, which was associated with the change in BP.

Compared with the control group, cardiac protein expression of *α*-MHC, COL1A1, and COL3A1 was substantially elevated in the SHR and 2K1C groups. Only in the 2K1C plus RDN group, RDN significantly attenuated protein expression of *α*-MHC, COL1A1, and COL3A1 when compared with the sham group (Figures 3(b), 3(c), 3(e), and 3(f)).

### 3.4. Effect of RDN on Kidney Remodeling

#### 3.4.1. Kidney Remodeling in SHRs

**Figure 4:**
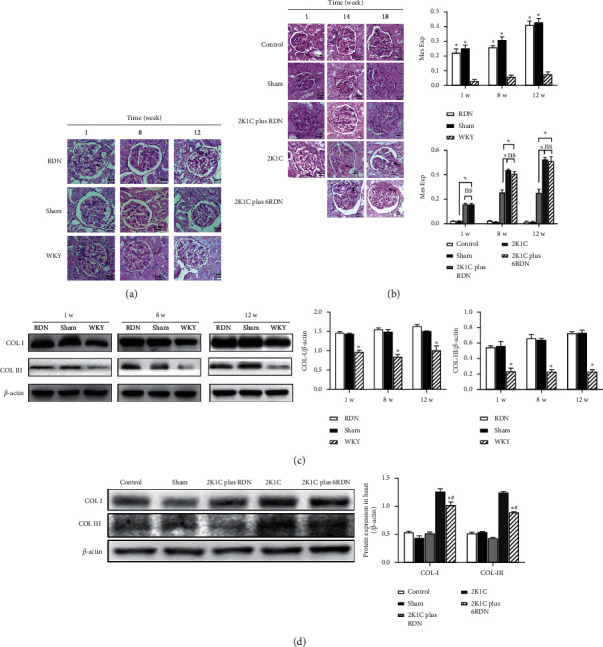
(a) Representative sections of kidney tissues with periodic acid-Schiff staining in SHRs. (b) Representative sections of kidney tissues with periodic acid-Schiff staining in SDs. (c) The protein expression and quantification of Col I and Col III protein in the kidney of SHRs. ^*∗*^*p* < 0.05. (d) The protein expression of Col I and Col III protein in the kidney of SDs. ^*∗*^*p* < 0.05 versus the control group (*n* = 4), sham group (*n* = 3), and 2K1C plus RDN group (*n* = 4). ^#^*p* > 0.05 versus the 2K1C group.

Reduction of renal sympathetic innervation in SHR rats by RDN was not associated with an attenuation of glomerular and tubulointerstitial injuries. Semiquantitative scoring of glomerular sclerosis revealed more pronounced glomerular damage in the sham group compared with the control. Immunohistochemical staining revealed pronounced podocyte damages in the sham group; however, this damage could not be reduced by RDN. Compared with the control group, renal gene expression of collagen I was substantially elevated in the sham group. RDN did not significantly attenuate gene expression of Col I when compared with the sham group (Figures 4(a) and 4(c)).

#### 3.4.2. Kidney Remodeling in SDs

Reduction of renal sympathetic innervation in the RDN group was associated with an attenuation of glomerular and tubulointerstitial injuries. Immunohistochemical staining showed striking podocyte damages in the sham group, which could be relieved only in the 2K1C plus RDN group rather than in 2K1C plus 6RDN rats. Compared with the control group, renal expression of collagen IA was substantially elevated in 2K1C and 2K1C plus 6RDN groups. RDN in the 2K1C plus RDN group significantly attenuated expression of Col IA, when compared with the 2K1C group (Figures 4(b) and 4(d)).

## 4. Discussion

The present study demonstrated that RDN performed before development of hypertension provided significant antihypertensive benefits in the secondary hypertension model. However, RDN could not maintain this antihypertensive effect in both rat models when hypertension had already developed. To our knowledge, the pathogenesis of hypertension is complicated, including primary hypertension and secondary hypertension. Many studies have confirmed that activation of the sympathetic nervous system and the renin-angiotensin-aldosterone system (RAAS) serve a critical role in regulating the blood pressure. However, the specific role the sympathetic nerves serve in these two systems is still not clear. In 2014, SYMPLICITY HTN-3 was used as the first double-blind randomized trial and it failed to reach its primary endpoint of the antihypertensive effect [8, 16], which has caused a decline in interest for RDN research. Nevertheless, the SYMPLICITY HTN-3 trial also had its own limitations; many clinical trials and animal experiments showed that the sympathetic nerve could not only modulate the transient blood pressure, but it could play an important role in long-term blood pressure control [17]. Therefore, understanding the role of the RDN procedure among animal models is of great importance. This was the first study to observe the effect of RDN on blood pressure and target organs in two types of hypertensive rat.

The spontaneously hypertensive rat (SHR) is an excellent animal model for experimental hypertension that could serve as a counterpart for clinical essential hypertension. The blood pressure of SHR increased as the rat aged, reaching 150 mmHg at five weeks old. The male SHR (age: 12 weeks) mean arterial pressure (MAP) averages approximately 170–180 mmHg. Many previous studies have confirmed the excessive activation of the sympathetic nervous, and RAAS systems served an important role in the development of SHR hypertension [18, 19]. Thus, SHR is the appropriate animal model for the study of primary hypertension. Conversely, the 2K1C rat model is similar to the pathogenesis of renal vascular hypertension in a human model [17]. Renovascular hypertension is the most common type of secondary hypertension. Therefore, the objective of this study was not only to evaluate the effect of RDN in different types of hypertensive rats (through the application of a simpler and more economic hypertensive animal model); the most important aim of this study was to evaluate the statuses and roles of the sympathetic nervous and RAAS systems comprehensively in different types of hypertension.

Many experiments have demonstrated that combined resection of the renal sympathetic nerve and chemical renal denervation downregulated the blood pressure by effectively suppressing the renal sympathetic nerve [18, 20, 21]. Previous studies have shown that RDN in 12-week-old SHR could effectively reduce blood pressure and help in maintaining a constant blood pressure at normal levels [22, 23]. However, some studies had reported that a transient blood pressure drop after 12 weeks of RDN is observed in SH rats, with blood pressure then returning to the preoperative level or a slightly elevated level [24, 25]. The result of this study showed that the blood pressure decreased significantly after one week of RDN; meanwhile, the concentrations of NE and Ang II in the plasma were significantly lower than those in the sham group, indicating that bilateral RDN was effective [26]. Subsequently, the BP increased gradually in the SHR RDN group, and eight weeks following the RDN operation, there was no significant difference in blood pressure between the SHR-RDN group and the SHR-sham group. During the entire experimental period, the concentrations of NE and Ang II in the SHR group were higher than those in same-age rats in the WKY group, indicating that the renal sympathetic nerve activity of SHR was in a continuous activation state. Interestingly, NE and Ang II levels in SHR reduced temporally but rose again after intervention of RDN, signifying that the RDN procedure inhibited NE release and the RAAS system to reduce blood pressure. However, this method could not achieve a lasting and stable antihypertensive effect. According to the above phenomena, it can be speculated that (1) the RAAS system is involved in the maintenance of hypertension in SHR, and (2) the RAAS system had been activated at the beginning of the increase in BP and maintained by the central sympathetic system, so RDN could not reverse central sympathetic activation. Even the local RAAS was modified by RDN, and the blood pressure could not continuously decrease due to the ineffectiveness of RDN on the central maintenance mechanism. In addition, previous studies found that renal sympathetic nerve regeneration occurs in the renal sympathetic nerve for 14 days. Thus, the results of this study do not rule out the possibility of neural regeneration [27].

Studies have shown that, in the renovascular hypertensive model, the blood pressure of the 2K1C rats increased rapidly, accompanied with an elevation of NE and Ang II in the plasma, four weeks postoperatively [16, 28]. The systolic blood pressure in the 2K1C rats elevated one week after surgery and was accompanied by the increase of NE and Ang II, indicating that the renovascular blood pressure model was successfully established. The blood pressure and NE and Ang II concentrations in the 2K1C plus RDN group were not significantly different from those of the sham group at the corresponding timepoints. These results suggest that a simultaneously operated RDN procedure before the establishment of a renovascular hypertension model may lead to the blood pressure near normal by inhibiting the excessive activation of the RAAS system. They may also suggest that the renal sympathetic nerve served an important role in the occurrence and development of renovascular hypertension. This is consistent with the results of Gouvea et al. who demonstrated that renal sympathetic activity plays a role in the genesis and the maintenance of hypertension [29]. However, the antihypertensive effect of RDN did not appear as expected in the 2K1C plus 6RDN group. Compared with the 2K1C group, the blood pressure of the group 2K1C plus and 6RDN only had a declining trend after the RDN procedure. The authors discussed two possible causes of this result: (1) the renal sympathetic nerve was activated after the 2K1C surgery, leading to activation of the systemic sympathetic system through brain central sympathetic nerve afferents. Therefore, the blood pressure could not drop six weeks after the RDN procedure due to the procedure only blocking the local sympathetic system and activating the brain central sympathetic nerve. (2) Likewise, sympathetic nerve regeneration must be taken into consideration. Mulder et al., 2013, demonstrated that, in normotensive rats, reinnervation of the renal sensory nerves occurs over the same time course as reinnervation of the renal sympathetic nerves, both being complete at nine to twelve weeks following RDN [30]. In transplanted rat kidneys, some reinnervation occurs within nine months after transplantation [31]. The two mechanisms mentioned above attenuated the benefit of RDN, and the specific mechanism needs to be confirmed by further experiments.

Previous studies have shown that hypertension can lead to myocardial hypertrophy, atherosclerosis, and fibrosis of the heart and kidneys [32–35]. The *α*-MHC level, as an index of degree of ventricular hypertrophy damage, is mainly in ventricular myocytes of adult rats. Persistence after load can induce transformation of *α*-MHC into *β*-MHC, resulting in decreased expression of *α*-MHC [36]. Several animal experiments and clinical trials have shown that RDN could significantly improve left ventricular hypertrophy induced by hypertension [36–38]. Col I, as an indicator of fibrosis, increased accordingly as heart or kidney fibrosis aggravated [39, 40]. This observation confirmed that the organ damage was induced by hypertension in the cardiovascular system and the kidney. Semiquantitative analysis of histological staining showed that the higher the blood pressure, the heavier damage in target organs, including myocardial hypertrophy, aortic remodeling, and renal fibrosis. Notably, only the 2K1C plus RDN rats demonstrated no impaired performance in target organs. RDN seemingly had no effect of preventing target organ damage in the presence of hypertension, either in the primary or secondary hypertensive model. Furthermore, even the blood pressure and hormone levels were reduced to normal levels in the SHR RDN group; the expected target organ benefit was not observed. Moreover, histological observations have been further confirmed by the detection of proteins in the heart and kidneys. This result may not be consistent with those of previous animal experiments.

A key limitation of our study was not recording systemic sympathetic activity in all groups. Moreover, additional limitations exist during the clinical treatment, for both the primary and secondary hypertensive models. Specifically, the models utilized in this study were already diagnosed with hypertension, and the RAAS system had been activated. Previous studies have confirmed the marginal effect of RDN on hypertension under the condition of RAAS already being activated. This condition was considered as one of the possible reasons for a clinically depressed outcome in the HTN-3 trial. However, it is notable that the 2K1C plus RDN group and early intervention on the SHR group showed significant effects on blood pressure. Moreover, the methods of how to maintain this effect and whether intervention time can be advanced still need further study. Therefore, a large number of clinical trials are needed to assess the practicality of early prophylactic ablation in patients with a family hypertension history or high risk of secondary hypertension.

## 5. Conclusion

Increased blood pressure is associated with RAAS activation, which likely leads to the cascade effect, an ongoing process that continues to increase blood pressure and, thereby, exerts sustained damage to target organs. This study confirms that RAAS activation is expected to have a positive effect on reducing blood pressure and preventing target organ damage if RDN blocks the local sympathetic nerve before systematic RAAS activation in both hypertensive models.

## Data Availability

The data used to support the findings of this study are available from the corresponding author upon request.

## Abbreviations

LV: Left ventricle
LVMI: Left ventricular mass index
MAP: Mean arterial pressure
NE: Norepinephrine
RAAS: Renin-angiotensin-aldosterone system
RDN: Renal denervation
RDN: Renal sympathetic denervation
RND: Renal nerve denervation
SBP: Systolic blood pressure
SD: Sprague–Dawley
SHR: Spontaneously hypertensive rats
WKY: Wistar–Kyoto.
